# Targeting cccDNA for Degradation: Endogenous- and Exogenous-Based Methods of Nuclear-Localized DNA Destruction in the Context of Chronic Hepatitis B Infection

**DOI:** 10.3390/v18090929

**Published:** 2026-08-24

**Authors:** Cassie Thompson, Vanessa Meier-Stephenson

**Affiliations:** 1 Department of Medical Microbiology and Immunology, University of Alberta, Edmonton, AB T6G 2R3, Canada; cjthomp1@ualberta.ca; 2 Department of Medicine, University of Alberta, Edmonton, AB T6G 2R3, Canada; 3 Li Ka Shing Institute of Virology, University of Alberta, Edmonton, AB T6G 2R3, Canada

**Keywords:** hepatitis B virus (HBV), cccDNA, endogenous DNA degradation, apoptosis, APOBECs, CRISPR

## Abstract

The ability to facilitate cellular degradation or achieve therapeutic destruction of targeted noxious DNA is recognized as a major goal for the treatment of various viral infections. In the case of chronic hepatitis B, targeted DNA degradation of the episomal viral genome found in the nucleus of infected hepatocytes is key to achieving a true cure. However, mechanisms of targeted DNA degradation without excessive broadscale cytotoxicity are still under investigation by researchers. This review summarizes several current systems known to have the ability to target nuclear-localized DNA, including endogenous methods of DNA degradation that may be harnessed therapeutically and exogenous methods such as CRISPR or other xenobiotics, with a focus on their potential application in hepatitis B treatment.

## 1. Introduction

Hepatitis B is a global health concern. In 2022, it was estimated that over 254 million people worldwide were infected with the virus responsible for the disease, the hepatitis B virus (HBV) [1]. Chronic HBV infection is one of the leading causes of hepatocellular carcinoma (HCC) and subsequent liver-related deaths worldwide [2]. If acquired in adulthood through contact with infected bodily fluids, the disease is typically acute with less than 5% of adult individuals developing chronic infection. Unfortunately, the most common route of transmission, from mother to child at birth (vertical transmission), coincides with the age at which acquiring chronic HBV is greatest (upwards of 95% in infected neonates) [3]. While an effective prophylactic vaccine exists and 190 nations participated in infant immunization as of 2024, infection is still prevalent in many regions [4].

**Figure 1:**
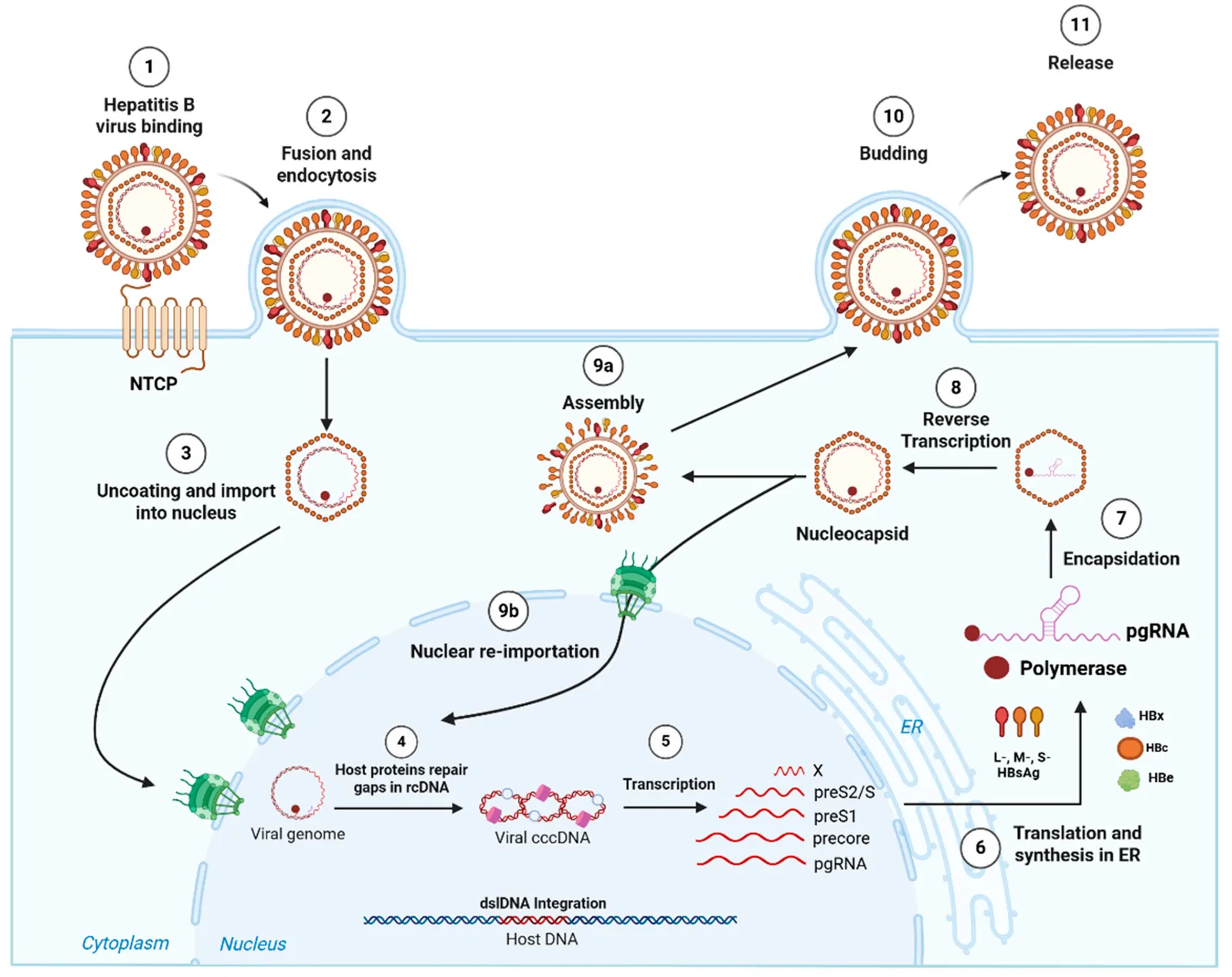
HBV replication cycle. Establishment of chronic HBV infection is dependent on the viral genome’s nuclear translocation and circularization into covalently closed circular DNA (cccDNA). Upon NTCP receptor binding (1) and receptor-mediated endocytosis (2), relaxed circular HBV DNA (rcDNA) from the viral capsid is imported into the nucleus (3). rcDNA then uses host cell DNA repair machinery to circularize into cccDNA (4), which is chromatinized to create the stable HBV minigenome. This form produces all the transcripts necessary for transcription (5). Translation of the mRNAs (6) is followed by pgRNA encapsidation once polymerase binds (7). Reverse transcription of pgRNA to rcDNA takes place inside the capsid (8) while the virion either continues to assemble its surface proteins for viral assembly, budding, and release (9a, 10, 11) or can undergo nuclear re-importation (9b) as a naked nucleocapsid form. Created in BioRender. Meier-Stephenson, V. (2026) https://BioRender.com/my07etv.

HBV is a partially double-stranded DNA virus that persists in host hepatocytes in a chronic episomal form. The lifecycle of HBV begins with cellular entry via association with the liver-specific sodium taurocholate co-transporting polypeptide (NTCP) receptor, enabling subsequent endocytosis (Figure 1) [5]. After translocation to the nucleus and nucleocapsid disassembly, relaxed-circular DNA (rcDNA) is released. Interaction with host DNA repair factors facilitates the conversion of the rcDNA into covalently closed circular DNA (cccDNA) [6,7,8] before the molecule undergoes the process of chromatinization [9]. This cccDNA acts as a viral reservoir, providing all the RNA transcripts, including the pre-genomic RNA (pgRNA), required for replication [10]. Upon translation of viral mRNAs, pgRNA transcripts destined to become new virions are bound by the polymerase and encapsidated. The pgRNA continues to be processed inside the capsid, forming either new rcDNA, or less commonly, a mis-primed double-stranded linear DNA (dslDNA) [11]. Nucleocapsids can then gather newly formed surface proteins to assemble, bud and exit the cell. Alternatively, nuclear re-importation can occur in an auto-amplification step, whereby new cccDNA molecules can be formed, allowing for stability of the cccDNA pool [6].

In the case of virions or nucleocapsids containing the mis-primed linear DNA forms, once inside the nucleus, these linear forms have the potential to integrate into the human genome at sites of DNA damage [11,12,13]. While incapable of replication, this integrated form is still able to produce viral surface proteins (HBsAg) and truncated forms of the X protein (HBx) [12], contributing to the persistence of serological HBsAg detection in chronic HBV infection [14,15] and the pro-inflammatory/carcinogenic effects attributed to HBx [16]. Furthermore, integrations can have profound impacts on the development of HBV-induced HCC including insertional mutagenesis and chromosomal instability [11]. Importantly, and in relation to this review, integrated forms complicate strategies for cccDNA clearance by exogenous methods as they represent an alternate target for any sequence-specific approach.

With regard to current treatments, the mainstay of treatment for HBV-infected individuals has been nucleos(t)ide analogs (NAs) [17,18]. NAs are able to suppress HBV replication but often require lifelong treatment [6]. Previously, pegylated interferon-α (IFNα) was also used, and although capable of inducing “functional cure” in select individuals (defined as sustained, undetectable HBV surface protein and HBV DNA in serum after completion of a finite course of treatment) [19], there is a high risk of side effects/low treatment tolerance. With both drugs, there is potential for relapse upon cessation due to incomplete viral clearance [6,18,20,21].

Although several other therapies such as entry inhibitors, antisense oligomers, and capsid inhibitors are now undergoing phase II and III clinical trials and are becoming more widely available [22,23,24,25], these strategies target processes downstream of the cccDNA; as such, the cccDNA will persist even in cases where patients reach “functional cure”. “True cure” or “sterilizing cure” is when all viral products have been eliminated—meaning all the cccDNA and integrated copies have been eliminated. While often deemed an unrealistic goal, we sought to focus on one part of this challenge—the cccDNA. In the following review, both the endogenous and exogenous strategies for destruction of cccDNA are reviewed, with a focus on mechanistic and safety considerations for potential future application in the treatment of chronic HBV.

## 2. Endogenous DNA Degradation Pathways

Endogenous processes exist to facilitate the destruction of nuclear DNA. Below, several examples of such DNA degradation pathways are highlighted, such as those that occur during apoptosis, during cell division, and after mutation via the actions of the APOBEC (apolipoprotein B mRNA editing catalytic polypeptide-like) family of cytidine deaminases. These methods are explored and further compiled for comparison (Table 1).

### 2.1. Apoptosis

The most common methods of endogenous DNA degradation are those carried out during the process of apoptosis. Apoptosis is an innate process involving the removal of damaged/dangerous cells in a controlled manner, during which chromosomal DNA is commonly targeted [26,27]. Apoptosis of an HBV-infected cell would promote destruction of all cell contents, including the viral cccDNA contained within. While various endonucleases have been identified as having roles in the processing of DNA during apoptosis (such as DNase γ [28]), the most prevalent endonuclease responsible for the cell-autonomous fragmentation of chromosomal DNA during apoptosis is the caspase-activated DNase (CAD), a nuclear localized enzyme [26]. Normally complexed with the inhibitor of CAD (ICAD), the CAD/ICAD complex is cleaved via caspase-3 during both extrinsic and intrinsic pathways of apoptosis, resulting in the enzymatically active CAD [29]. Freed CAD then proceeds to form a “scissor-like” homodimer and induce internucleosomal fragmentation [30]. However, CAD cleaves DNA with minimal sequence specificity for genomic targets and would therefore not allow for selective destruction of noxious DNA such as the cccDNA [31].

Apoptosis-inducing agents have been briefly investigated with regard to HBV. After the discovery that mice deficient in inhibitors of apoptosis proteins (IAPs), a class of negative apoptotic regulators in mammalian cells [32], showed decreased markers of HBV [33], researchers used small-molecule inhibitors of IAPs to investigate the potential of these drugs for promoting HBV clearance [34]. Administration of IAP inhibitors resulted in suppression of serum HBV-DNA to undetectable levels in an immunocompetent HBV mouse model compared to vehicle controls. In addition, only transient elevations in hepatic injury markers were noted after administration of the inhibitors, with markers returning to baseline within 48 h indicative of limited liver injury sustained. Although treatment caused a 35-fold reduction in HBV genomes in infected mice compared to in controls, residual HBV was still identified after treatment conclusion [34]. One of the investigated small molecules, birinapant, reached phase I trials for use in HBV (NCT02288208); however, the trial was terminated due to the observance of cranial nerve palsies. While safety profiles must be further established for this class of drug to prevent exacerbation of liver injury seen in many chronic HBV cases, other IAPs are being investigated for use in the treatment of HBV (NCT04568265) [35].

HBV also has the ability to influence apoptotic signaling via its X protein, HBx, which has been shown to have both pro- and anti-apoptotic mechanisms [36], the details of which must be better understood prior to use of apoptosis-inducing agents. The above IAP preclinical data showed promise for minimal destruction of bystander cells, but it remains a possibility that other apoptosis-inducing agents may not be as controlled and could potentiate or exacerbate pre-existing liver injury. Factors, such as interindividual variability with regard to disease progression [37], potential side effects, and the lack of ability to target infected cells, and the induction of apoptosis as a method of clearing HBV infection, would require further research.

### 2.2. Cell Division and Cytoplasmic Destruction of Nuclear DNA

Upon nuclear DNA damage, an accumulation of cytoplasmic DNA can occur in the form of micronuclei. These small satellite organelles contain circular host DNA and are products of chromosomal damage mis-segregation during cell division [38]. Micronuclei have been shown to frequently undergo irreversible nuclear-envelope collapse, exposing the contained DNA to the cytosol [39]. This cytosolic DNA can then be degraded by exonucleases (such as TREX1 [40]) or act as a danger-associated molecular patterns (DAMPs), resulting in immunogenic signalling via sensors such as cyclic GMP-AMP synthase (cGAS) and the subsequently activated cyclic GMP-AMP receptor stimulator of interferon genes (STING) [38]. Although HBV cccDNA resides intranuclearly, there is potential for cccDNA exposure to cytosolic DNA-sensing and destruction by these processes during the nuclear envelope breakdown seen in mitosis. Indeed, several studies have identified both in vitro and in vivo loss of cccDNA after the induction of cell division, leading to suggestions that cccDNA loss after mitosis of an infected cell may be due to mechanisms similar to those involved in the host cytoplasmic DNA degradation [41,42,43].

Unfortunately, two issues emerge when considering the utility of cell division as a strategy for clearing HBV infection. The use of agents to induce cell division brings about the risk of both liver decompensation and carcinogenicity [42]. Additionally, complete viral clearance was not achieved in the in vivo studies, even with rates of division likely far exceeding those seen in humans. This was hypothesized to be due to the persistence of non-proliferating infected hepatocytes, with indications that cells expressing high HBV levels may have reduced replicative abilities when compared to uninfected hepatocytes [41]. These non-replicating cells may act as reservoirs for the virus, interfering with the success of cell division-based strategies in clearing HBV infection.

Recently, a process called macronucleophagy has been identified in both yeast and mammals [44]. Macronucleophagy, a form of autophagy (from the Greek “self” and “eating”) is a process whereby specific nuclear components are sequestered within the autophagosome to undergo degradation [45]. Nuclear lamins have been shown to be targeted for macronucleophagy [46], as well as SIRT1 [47], an NAD+-dependent deacetylase that has been identified as being associated with HBV-induced HCC [48]. Interestingly, with regard to cccDNA targeting, colocalization of DNA with the autophagosomal marker LC3 and the lysosomal protein LAMP1 [49], as well as the visualization of nuclear-to-cytoplasmic chromatin blebbing [50], points towards a nuclear-to-autophagosome-to-lysosome transport pathway that targets nuclear DNA for degradation. The specific mechanisms of macronucleophagic targeting are not currently well understood, with the only identified initiators of nucleophagy being DNA damage and senescence [49,50]. While macronucleophagy has not been investigated with regard to HBV, it remains a novel method of cell-driven DNA removal, the mechanisms of which, including targeting, localization, and efficiency of degradation, require identification to determine the viability of usage in an HBV model. Once established, the next challenge is to identify ways to utilize the system for removal of cccDNA.

Although future research is needed to characterize the specific pathways used in targeting proteins for macronucleophagy, autophagy, in general, is emerging as a contributing component of cellular antiviral defense against certain viruses [51]. It has been identified that certain alkaloid compounds can induce selective autophagy of the HBV core protein, causing dose-dependant reductions in HBV [52]. However, previous studies have shown that HBV also has the capability to induce early autophagy to allow for increases in viral replication. Inhibition of the autophagosome caused marked decreases in HBV production and has been shown to impair HBV core protein assembly and viral egress from hepatocytes [53]. This counterintuitive induction by the virus may then have other potential effects that must be weighted when considering autophagy as a method of viral clearance [54,55]. While there is potential of harnessing the antiviral capabilities of the host cells autophagosome for degradation of HBV, the relationship between the virus and host systems must be established before therapeutic targeting can be achieved.

### 2.3. APOBEC Cytidine Deaminases

The APOBEC family, consisting of 11 polynucleotide cytidine deaminases [56], is responsible for mediating deamination of cytosines to uracil, causing C:U mutations at targeted sites [57]. These enzymes have been identified as having roles in physiological processes such as lipid metabolism [58] and the regulation of cellular innate and adaptive immune responses to pathogens [59]. When looking specifically at the role of these enzymes in viral DNA degradation, APOBEC3 enzymes (A3’s) A3A–A3H (seven proteins: A3A/B/C/D/F/G/H) interact with single-stranded DNA (ssDNA) through nucleophilic attack to mediate the deamination, resulting in the C:U conversion [56]. This process of deamination can then result in hindered viral reverse transcriptase function, lingering DNA mutations, or direct degradation of the viral mutated DNA through abasic site cleavage by endonucleases [59,60]. A3G was first identified as having antiviral effects against human immunodeficiency virus-1 (HIV-1), [61] and since then, other A3’s have been found to act as part of the innate immune defence against a range of viruses, including HIV-1, human papillomavirus (HPV), and HBV [62].

Endogenous targeting of APOBECs to DNA is due to enzyme preferences for specific ssDNA dinucleotides (such as A3G targeting cytosine bases preceded by another cytosine (5′-CC)), as well as ssDNA secondary structures, such as the loop of stem-loop ssDNA being preferentially targeted by A3G in the case of HIV-1 cDNA [63,64]. Recently, it has been discovered that cofactors used by APOBEC1 may also have roles in regulating the A3s mutational activity [65]. Most notably in the case of HBV is the role of hnRNPA2B1, a member of the heterogenous nuclear ribonucleoprotein family (hnRNPs). Acting as a nuclear DNA interacting protein, hnRNPA2B1 has been found to have a role in both the regulation of genome stability [66] and the type I interferon response after detection of viral DNA fragments [67]. hnRNPA2B1 has also been identified as a cccDNA-binding protein that recruits A3B to trigger deamination of cccDNA [68], with one in vivo study showing that an agonist of hnRNPA2B1 significantly reduces cccDNA levels in liver tissues [69].

If enough mutations are induced in DNA, the DNA may be marked for degradation rather than repair [60]. Leaning on these discoveries, efforts to utilize APOBECs for HBV clearance have been made in recent years. IFN-α can cause the expression of A3B, A3F, and A3G in hepatocytes, with deamination of viral intermediates (including cccDNA) potentially explaining the viral suppression seen from IFN-α therapy [70]. A3G has been shown in vivo to edit up to 35% of viral genomes found in human cirrhotic tissue [71]. Interaction of viral DNA with A3s has resulted in decreases in cccDNA, as well as lower levels of HBV replicative intermediates (rcDNA or pgRNA) [68,72,73,74,75]. However, the precise mechanism of APOBEC-induced reductions in cccDNA and viral pgRNA is still a topic of debate. While research has indicated that A3A and A3B are able to deaminate nuclear-localized cccDNA [73,75], it has also been shown that A3C, A3D, and A3H are able to exert antiviral activity without cccDNA deamination, leading to the conclusion that these specific APOBEC enzymes have a complex influence on intracellular processes, resulting in viral inhibition rather than direct deamination of viral cccDNA [76]. It has also been identified that A3B has the ability to interact with the HBV core protein to edit HBV core-associated DNA, indicating potential inhibitory activity during reverse transcription in addition to effects on the cccDNA [77]. Given that A3s are known to mainly interact with ssDNA, it has been shown that the single-stranded region of the rcDNA HBV intermediate is a natural target for APOBECs [59]. Although this does not explain the decreases seen in the double-stranded cccDNA after exposure to A3s, it has been proposed that nuclear-localized A3A and A3B act on cccDNA when it is transiently present in its ssDNA form prior to transcription initiation [78,79]. Indeed, A3A has been shown to only act on transcriptionally active cccDNA [80]. A3B may act in a transcription-independent manner [72]. Further research is required to identify the specific mechanisms by which each of these enzymes affects HBV cccDNA, with replication and validation of findings regarding the extent of APOBEC-mediated deamination of cccDNA representing a necessary step towards understanding their utility in an anti-HBV context.

While the effects of APOBEC-mediated degradation of cccDNA are highlighted for their ability to recognize foreign DNA, the use of these enzymes to therapeutically induce HBV DNA degradation may not be possible due to the scale of these enzymes’ mutational capacity with regard to the host genome. Studies have confirmed that dysregulation and an overabundance of A3s can result in genomic instability and potential carcinogenesis [81,82]. The somatic mutation spectra dominated by C-to-T transitions seen in many cancers are likely caused by A3B, with tumors that express high levels of A3B having twice as many mutations as those that express low levels of A3B [83]. Using an in vivo mouse model, it was noted that high and sustained levels of A3B compromises animal survival by inducing DNA damage and inflammation [84]. Overexpression of A3B has also been identified in cases of HCC, correlating with the ratio of mutations observed and fostering tumor occurrence and metastasis in vivo [79], as well as overall disease progression [85].

In HBV, the APOBEC family therefore has a dual role. These enzymes have been identified to both directly cause cccDNA degradation and inhibit HBV replication, potentially leading to decreases in inflammatory reactions and progression of disease. However, APOBECs are also known to target the host genome, occasionally causing host genome instability and increased mutation risk. As such, overexpression of APOBECs and/or induction of their recruitment should be considered with caution as a strategy for HBV clearance.

### 2.4. Immune Modulation

Host immune responses play a prominent role in HBV control and pathogenesis during infection. In acute HBV infections, both cytolytic and non-cytolytic immune responses are noted. Destruction of infected hepatocytes, as evidenced by flares of liver injury markers, has been found to be predictive of HBsAg loss [86]. Non-cytolytic viral suppression by CD8+ cells can also occur [87,88,89] and is mediated through the release of cytokines, namely interferon-γ (IFN-γ) and tumor necrosis factor-α (TNF-α) [90]. Production of these cytokines from T-cells has been shown to induce cccDNA deamination and decay via up-regulation of APOBEC3A and APOBEC3B, resulting in loss of cccDNA [75]. While the mechanisms of acute control of HBV infection have yet to be fully understood, a combination of both cytolytic and non-cytolytic immune responses appears to be needed for resolution of infection.

In cases of chronic HBV, immune dysregulation is observed both systemically and in the liver microenvironment, with innate and adaptive immune cells showing dysfunctional phenotypes and abnormalities in response [91]. The noted abnormalities include dysfunctional HBsAg-specific B-cells [92], delayed or absent antibody responses [93], or HBV-specific T cell exhaustion [94]. Details of immune dysregulation in chronic HBV infection will not be extensively discussed in this review (see [87,91] for a more in-depth analysis).

Given the insufficient immune response to chronic HBV, a number of researchers are investigating immunomodulators and therapeutic vaccines to stimulate a more robust response. While many of these treatments have the goal of a functional cure rather than destruction of cccDNA, the mechanism of immune stimulation has been shown to result in cccDNA degradation via both cytolytic and non-cytolytic responses noted above.

Toll-like receptor (TLR) agonists stimulate the innate immune system, leading to the production of cytokines such as TNF-α and IFN-γ and indirectly promoting HBV-specific T-cell responses [91,95]. Several of these agonists have reached phase II clinical trials (NCT03615066, NCT02579382, and NCT05551273). Programmed death-1 (PD-1) and PD-1 ligand (PD-L1) inhibitors re-initiate anti-viral responses via reversal of HBV-specific T-cell exhaustion, through targeting PD-1 inhibitory receptors and ligands responsible for T-cell suppression [96]. A phase IIb trial of one such PD-L1 inhibitor has been completed (NCT04465890) [97].

Therapeutic vaccines aim to prompt a new or enhanced immune response to pre-existing conditions through mechanisms such as de novo priming of T-cells or rescuing dysfunctional T-cell response [98,99]. Therapeutic vaccines consist of vectors containing incorporated HBV antigens such as HBV core antigen (HBcAg) and HBsAg, with the goal of evoking new or amplifying existing anti-HBV immune responses [98,100]. Several therapeutic vaccines have reached clinical trials [101,102] (NCT03866187); however, while some appear to restore T-cell-mediated cytokine release, the response alone is insufficient to reach trial endpoints of HBsAg loss and will likely require combination therapy to reduce circulating HBV antigens to facilitate reversal of immune exhaustion (also currently being trialed—NCT04749368) [91,100].

## 3. Exogenous DNA Degradation Strategies

Newer methods of DNA targeting using exogenous factors and therapeutics are beginning to be applied for treatment of HBV. The discovery of clustered regularly interspaced short palindromic repeats (CRISPR), as well as anti-cancer agents that are able to bind and destroy DNA, show potential as a way to target cccDNA directly. Strengths and limitations of these methods are explored below and in Table 1.

### 3.1. CRISPR/Cas9

A major development in the ability to target specific DNA sequences has been the discovery and implementation of the CRISPR/Cas9 genomic-editing technology. Biologically originating from the adaptive immune system of prokaryotes [103], CRISPR/Cas9 enables researchers to selectively edit genomic sequences and has become widely used in the field of genetic engineering [104]. In bacteria, adaptive immunity against foreign DNA is acquired by integrating fragments of foreign DNA into CRISPR arrays [105]. After transcription of the CRISPR array, complexing of the array with a trans-activating CRISPR RNA (tracrRNA) and the Cas9 endonuclease occurs, creating a ribonucleoprotein (RNP) complex [106,107]. Upon foreign DNA reinfection, complex recognition of the foreign DNA occurs and causes double-stranded cleavage of the target by Cas9 [108].

Recently, optimization of this system has resulted in the requirement of only two major components; a Cas endonuclease and a gRNA (guide RNA, a fusion of the transcribed CRISPR array and the tracrRNA) designed against the targeted sequence [104,106]. Double-stranded breaks produced by Cas9 at targeted regions can trigger gene knockout via frameshift mutations or be used in programmable genome insertions, leading to CRISPR being attractive for treatment of cases such as genetic diseases [109,110,111] or antiviral treatment [112,113,114]. Using gRNAs targeting various regions of the HBV genome, CRISPR gene editing has shown a combination of deletion and insertion mutations in the HBV DNA [115], as well as significant inhibition of HBV protein production [114,116]. cccDNA is shown to be decreased or mutated to various degrees using these targeted gRNAs [114,116,117]. Further development of a Cas9 endonuclease resistant to host cell degradation has resulted in greater clearance of the HBV genome in vitro [118]. While it has been noted that many studies involving CRISPR in regard to HBV use models involving intracellular overexpression of CRISPR-Cas from plasmid vectors, which does not allow for direct clinical translation, others have remediated that issue via development of a single, transient delivery of CRISPR-Cas9 RNPs [119]. This method therefore allows for more therapeutic translation, with results showing over 98% HBV cccDNA degradation.

Although CRISPR remains a promising technique with regard to antiviral therapeutics, major limitations have been noted when using the technique in the clearance of viral cccDNA. Firstly, while CRISPR can be highly effective in eradicating cccDNA [119], viral rebound is noted to occur even after near-complete eradication of the cccDNA. This rebound is likely due to nuclear reimportation of rcDNA and pgRNA [120]. The observed viral rebound indicates that any treatment targeting cccDNA allows for complete degradation of all cccDNA molecules. The potential for mutation rather than degradation of the damaged cccDNA is also a concern, with the fate of cccDNA after CRISPR targeting still debated [121]; it has been observed that transcriptionally active episomal HBV variants appear after CRISPR treatment [122]. In light of this finding, some researchers have sought to deplete the host system likely responsible for repair of the DSBs induced by CRISPR in the HBV genome [123,124]. However, these studies have produced conflicting results regarding the benefit of this strategy. In addition, introducing double-stranded breaks into the cccDNA has the possibility of allowing further integration, similar to that of the integration of double-stranded linear DNA generated during new infection [122]. CRISPR has also been shown to have the potential to cleave at non-target sites of partial sequence homology, resulting in insertions, deletions, or chromosomal rearrangements, posing safety concerns over use therapeutically [125].

Further complicating the use of CRISPR in the treatment of chronic HBV is the integrated form of the HBV genome that is present in many infected cells alongside cccDNA [11]. Due to the sequence-based specificity of these enzymes, any gRNA targeting HBV will target both cccDNA and any integrated copies. This has the potential of inducing double-stranded breaks in any HBV that is integrated in the host genome, causing genomic instability. Further increasing the carcinogenicity of the virus may not outweigh the benefit of clearing the cccDNA.

Lastly, one major challenge that has recently emerged when aiming to use CRISPR therapeutics in clinical settings is the delivery of CRISPR components to target cells and tissues. Gene-editing efficiency depends highly on component transport to the cell nucleus, with different delivery techniques influencing specificity, efficiency, and overall therapeutic potential [126]. Challenges include tissue- and cell membrane-specific barriers [127], with other factors, such as Cas9 aggregation and immunogenic response to delivery vectors, presenting major hurdles to widespread implementation [126,128,129]. Viral vector-based delivery systems, such as adenoviruses, lentiviruses, and adeno-associated viruses, are employed in many of the United States Food and Drug Administration (FDA)-approved gene therapies [130]. However, viral vectors have major limitations, including high immunogenicity, risk of insertional mutagenesis, and limited cargo capacity of viral vectors [131,132,133,134]. Non-viral delivery systems such as lipid nanoparticles (LNPs) are noteworthy for their ability to enhance cellular uptake and promote stability and persistence of the CRISPR molecules in the cell, while avoiding restrictions of viral-based vectors [135]. Although they show promise in the field of gene therapy, challenges in clinical LNP usage include immunogenicity from different LNP formulations, as well as tissue-specific accumulation [136]. One important consideration is the hepatic accumulation observed when LNPs are delivered intravenously due to spontaneous absorption of ApoE on the surface of LNPs [137]. While some note this as a major limitation of LNPs [138], others have further modified LNP formulations to promote hepatic accumulation, resulting in efficient targeting of hepatic tissues [139,140]. This inherent liver-targeting positions LNPs as a promising platform for HBV treatment delivery [141].

### 3.2. Other Exogenous Endonucleases

Although Cas9 is the most commonly used genomic editor [142], other endonucleases have been investigated for application in anti-HBV targeting. Cas12a and Cas 12b, endonucleases resembling Cas9, have been used in CRISPR-based HBV diagnostics [143]. Recently, one in vitro study evaluated CRISPR/Cas12a in targeting plasmid HBV DNA; successful targeting and cleavage of HBV-DNA was achieved [144]. Future investigation is required to understand the impacts of these endonucleases on direct cccDNA degradation in more translational models.

Endonucleases which are not associated with the CRISPR technology are also used in gene editing, such as zinc finger nucleases (ZFNs) and transcription activator-like effector nucleases (TALENs). These endonucleases have the ability to induce site-specific double-stranded breaks and have been shown to suppress HBV replication [145]. Another class of endonucleases used in HBV targeting is the ARCUS nucleases. Engineered variants of the I-CreI homing endonuclease found in the algae *Chlamydomonas reinhardtii*, one ARCUS endonuclease resulted in an 85% reduction in HBV cccDNA in HBV-infected primary human hepatocytes (PHHs) [146]. Since this discovery, Precision BioSciences has initiated a phase 1 clinical trial of PBGENE-HBV (NCT06680232), an LNP-encapsulated mRNA encoding for an ARCUS endonuclease [147]. Initial data from a small cohort of patients in late 2025 showed decreased HBsAg markers following administration of PBGENE-HBV [147]. While only 10 patients were included in this early cohort, the results are promising with regard to the use of endonuclease-based cccDNA-targeting agents in treatment of chronic HBV.

### 3.3. Chemotherapeutics

The first anti-cancer agents were discovered in the 1940s by Goodman and Gilman [148]. These preliminary treatments consisted of alkylating agents, a class of chemotherapeutic that induces cytotoxic effects through DNA damage. Reacting with nucleophilic moieties of DNA [149], alkylating agents can act through either alkylation or crosslinking of DNA, preventing replication or transcription and leading to fragmentation and mutation of the DNA [150]. Further advancements in the pharmacological treatment of cancer resulted in the development of various other drug classes, including but not limited to topoisomerase inhibitors, antitumor antibiotics, antimetabolites, monoclonal antibodies, and hormonal agents [150,151]. Each class of anti-cancer chemotherapeutic has distinct mechanisms, making them useful for targeting and destroying cancerous cells.

It must be noted that while cancer and viral infections vary in many ways, at the core of each disease is the dysregulation of DNA. As such, many anti-cancer medications have a mechanism that results in the destruction of cancerous DNA. It has been identified specifically that host cell topoisomerase enzymes are necessary for the synthesis of the HBV cccDNA, with topoisomerase I and II inhibitors significantly reducing amounts of cccDNA [152]. Daunorubicin, a clinically used anti-cancer agent, is a topoisomerase II inhibitor that has also been shown to initiate an anti-HBV response through activation of the cGAS-dependent innate immune response [153]. This innate immune response is typically activated through the recognition of cytosolic DNA and results in a type I interferon response through the cGAS-STING signalling pathway [154]. Similar to that of autophagic signalling, HBV has the ability to downregulate cGAS-STING to aid in immune evasion [155]. Circumventing this immune evasion using cGAS-STING-stimulating therapeutics, such as daunorubicin, represents a potential anti-HBV strategy.

However, while the potential of using already available DNA-targeting therapeutics in the treatment of HBV may seem appealing in clearing the virus, the use of these chemotherapeutics may result in more harm to patients undergoing treatment, both due to the effects of the therapy on the viral infection and the side effects associated with anti-cancer treatment. The first concerning factor is that these anti-cancer agents induce apoptosis in rapidly dividing cells [156]. This fact presents a two-sided problem: uncontrolled hepatocyte apoptosis culminating in liver damage is not desirable in the treatment of HBV infected individuals, and hepatocyte proliferation in the context of HBV is poorly understood. While the liver has the profound ability to regenerate itself, it has been identified that approximately 40% of hepatocytes do not undergo the process of cell division during liver regeneration following liver resection [157], and as such, HBV would persist in these non-replicating cells, as has been previously described [41]. Therefore, this would not allow for efficient targeting of HBV-infected hepatocytes by anti-cancer therapeutics, while still allowing for the severe systemic side effects associated with chemotherapy [158].

The second factor discouraging the use of anti-cancer agents in the treatment of HBV is the off-target effects on the immune cells, which simultaneously act to control HBV infection. With the use of chemotherapies, there is the potential for viral flares and viral reactivation (HBVr) in both chronically infected patients and functionally cured patients who were placed on a chemotherapeutic treatment regimen. In these patients, HBVr results in increases in morbidity and mortality [159,160,161,162]. Although the cause behind this reactivation is not fully known, it has been suggested that the immunosuppressive nature of many chemotherapies is the cause, with the level of immunosuppression and the intensity of treatment affecting both the risk and severity of HBVr [160,163]. This is especially concerning given the association between HBV infection and disease progression to HCC, as HBVr in cancer patients is linked with poor clinical outcomes, due to both treatment delays/termination [164] and overall disease-free states and overall survival [165]. Increased understanding as to the mechanism of HBVr and which treatment options are most/least likely to cause HBVr is needed. The lack of chemotherapeutic options for cancer patients who have had previous/have chronic HBV infection highlights the urgent need for an HBV cure.

**Table 1:** Summary of methods for nuclear-localized HBV cccDNA degradation.

Technique	Mechanism of DNA Degradation	Selectivity	Efficacy	Safety	Translational Stage	References
Apoptosis (Section 2.1)	Overall cell death, including destruction of cellular contents.	No selectivity for cccDNA; potential selectivity for HBV-infected hepatocytes.	Significant reduction in serum HBV-DNA in mouse models	Varied depending on the method of induction and drug variety, although severe adverse effects have been observed, with one clinical trial terminated due to safety concerns.	In vivo, with several candidates reaching clinical trials (NCT02288208 and NCT04568265)	[34,35,166]
Cell division and cytoplasmic destruction (Section 2.2)	Exposure of DNA to cytoplasmic DNA-sensing enzymes/phagosomal degradation	Unknown; potentially selective	Unknown; significant reductions observed in in vitro and in vivo models.	Initial in vivo data observed; no significant liver injury after cccDNA loss.	In vitro and in vivo	[41,42,43]
APOBEC cytidine deamination (Section 2.3)	Extensive mutagenesis resulting in damage recognition and cell-mediated destruction	Targets cccDNA. Host restriction factors may be used to generate a targeted antiviral deamination complex on the cccDNA minichromosome.	Significantly reduced cccDNA levels in liver tissue of infected mice.	No toxicity observed in in vitro and in vivo assays. Risks of adverse editing events at unintended sites, risk of host genomic instability due to HBV-integrated copies.	In vitro and in vivo	[60,68,69,73]
Immune modulation (Section 2.4)	Restoration of anti-HBV immune response	Varied; non-specific towards cccDNA, but may be selective for promoting destruction HBV-infected cells	Currently there is limited efficacy in clinical trial endpoints of HBsAg reduction.	Adverse effects reported such as nausea, vomiting, and chills. Risk of immune overactivation.	Phase II clinical trials (NCT03615066, NCT02579382, NCT04465890, and NCT03866187)	[75,91,101,102,167,168]
CRISPR (Section 3.1)	Induction of double-stranded breaks or extensive mutagenesis, resulting in damage recognition and cell-mediated destruction.	Chosen target sequences can be recognized. Target site selection may be restricted to sites containing specific sequences.	>98% reduction of HBV cccDNA in vitro	Risks of adverse editing events at unintended sites, risk of host genomic instability due to HBV-integrated copies.	In vitro and in vivo	[119,143,169,170]
Other exogenous endonucleases (Section 3.2)	Induction of double-strand breaks or extensive mutagenesis, resulting in damage recognition and cell-mediated destruction.	Chosen target sequences can be recognized. Target site selection may be restricted to sites containing specific sequences.	In vivo cccDNA surrogate editing or degradation of 83% compared to controls. cccDNA elimination rates in clinical trial pending.	Adverse effects such as hypotension and liver transaminase elevation observed; no dose-limiting toxicities reported. Risks of adverse editing events at unintended sites, risk of host genomic instability due to HBV integrated copies.	Phase I clinical trials (NCT06680232)	[146,147]
Chemotherapeutics (Section 3.3)	Various, including stimulation of cellular anti-HBV immune responses	Non-selective	Significantly reduced the amount of in vitro cccDNA	Risks of HBVr; side effects associated with chemotherapies.	In vitro	[152,153,158]

## 4. Concluding Remarks

While therapeutics such as NAs and IFNα have shown successes in controlling infection, the lack of treatments that can directly target the persistent cccDNA has prevented full clearance of the virus. While elimination of cccDNA alone does not equate to “sterilizing cure”, it would prevent any further infection of hepatocytes and new integrations as only the cccDNA (and not the integrated copies) can produce pgRNA transcripts that become new virions. This review has summarized methods that are currently capable of destructive elimination of HBV cccDNA and evaluated the progress and challenges that prevent implementation of the strategies.

One of the major challenges is achieving safe and selective targeting of cccDNA while avoiding damage to the host. The strategy carries risks of destruction to healthy bystander tissues from overactive responses to therapies, as well as unintended DNA editing at integration sites. Therefore, many of the techniques summarized for viral HBV elimination have associated risks that must be further evaluated prior to clinic use.

In response to these challenges, alternate approaches to achieving the same phenotypic outcome, namely inhibition of viral replicative ability, include non-destructive methods of eliminating HBV viral replication via cccDNA, such as epigenetic silencing or gene editing [171,172]. Relying on inhibition of key regions of the HBV genome rather than direct degradation, these approaches prioritize a functional cure endpoint rather than cccDNA destruction. The use of CRISPR or CRISPR interference (CRISPRi) [107] to silence key parts of the genome has been noted as potential strategies [173], with one such CRISPRi-based therapeutic in initial clinical trial stages (NCT07200193). Other strategies of gene editing include approaches that do not induce double-stranded breaks, such as utilizing APOBEC cytidine deaminase activity in combination with CRISPR technology in base-editing techniques [121,174]. While beyond the scope of this review, we would like to direct the reader to some recent informative reviews on these topics [175,176,177].

Whether through depletion, silencing, or direct degradation of the HBV cccDNA, a safe and effective method of definitively arresting HBV viral transcription and replication is necessary to establish a cure for chronic HBV infection.

## Data Availability

No new data were created or analyzed in this study. Data sharing is not applicable to this article.

## Abbreviations

The following abbreviations are used in this manuscript:

HBV	hepatitis B virus
HCC	hepatocellular carcinoma
NTCP	Na+-taurocholate co-transporting polypeptide
rcDNA	relaxed circular DNA
cccDNA	covalently closed circular DNA
pgRNA	pregenomic RNA
mRNA	messenger RNA
dslDNA	double-stranded linear DNA
HBsAg	HBV surface antigen
HBx	hepatitis B X protein
IFNα	interferon α
APOBEC	apolipoprotein B mRNA editing enzyme, catalytic polypeptide
CAD	caspase-activated DNase
ICAD	inhibitor of caspase-activated DNase
DAMPs	damage-associated molecular patterns
cGAS	cyclic GMP-AMP synthase
STING	stimulator of interferon genes
TREX1	three prime repair exonuclease 1
SIRT1	sirtuin-1
LC3	microtubule-associated proteins 1A/1B light chain 3B
LAMP1	lysosomal-associated membrane protein 1
ssDNA	single-stranded DNA
HIV-1	human immunodeficiency virus 1
HPC	human papillomavirus
cDNA	complementary DNA
hnRNPA2B1	heterogeneous nuclear ribonucleoproteins A2/B1
CRISPR	clustered regularly interspaced short palindromic repeats
tracrRNA	trans-activating CRISPR RNA
RNP	ribonucleoprotein
gRNA	guide RNA
FDA	United States Food and Drug Administration
LNP	lipid nanoparticle
ZFNs	zinc finger nucleases
TALENs	transcription activator-like effector nuclease
PHHs	primary human hepatocytes
HBVr	hepatitis B virus reactivation
IFN-γ	interferon-γ
TNF-α	tumor necrosis factor-α
TLR	Toll-like receptor
HBcAg	HBV core antigen
CRISPRi	CRISPR interference
